# Concordance of oral HPV prevalence between patients with oropharyngeal cancer and their partners

**DOI:** 10.1186/s13027-016-0066-9

**Published:** 2016-04-27

**Authors:** Anne S. Tsao, Vassiliki Papadimitrakopoulou, Heather Lin, Ming Guo, J. Jack Lee, F. Christopher Holsinger, Waun Ki Hong, Erich M. Sturgis

**Affiliations:** Departments of Thoracic & Head and Neck Medical Oncology, Unit 432, The University of Texas MD Anderson Cancer Center, 1515 Holcombe Blvd., Houston, TX 77030 USA; Biostatistics, The University of Texas MD Anderson Cancer Center, Houston, TX 77030 USA; Pathology, The University of Texas MD Anderson Cancer Center, Houston, TX 77030 USA; Department of Otolaryngology Head/Neck Surgery, The Cancer Center and School of Medicine Stanford University, Stanford, CA 94305 USA; Division of Cancer Medicine, The University of Texas MD Anderson Cancer Center, Houston, TX 77030 USA; Department of Head and Neck Surgery, The University of Texas MD Anderson Cancer Center, Houston, TX 77030 USA

**Keywords:** HPV, Partner transmission, Oropharyngeal cancer

## Abstract

**Background:**

Human papilloma virus (HPV) is a known causative factor in oropharyngeal squamous cell cancer (OPC). In this prospective study, we sought to define the risk of HPV transmission between OPC patients and their sexual partners by performing HPV genotyping on oral cytology brushings.

**Methods:**

Newly diagnosed OPC patients and their sexual partners underwent oral mouth swabs and answered a risk factor questionnaire. Patient tumor samples and oral swabs from both the patient and partner were assessed for HPV status and genotyped using Easy-Chip HPV Blot PCR.

**Results:**

We enrolled 227 patient-partner pairs and obtained sufficient analyzable DNA from both members in 198 pairs. Of 144 patients with available OPC tumor tissue, 128 (89 %) had HPV-positive tumors by either in situ hybridization or p16 immunohistochemical analysis (104 or 121, respectively). In total, there were 28 patients and 30 partners who were HPV positive by oral swab. The prevalence rate of oral HPV in partners was 15 %. There were 39 patient-partner pairs who had one or both members returning positive for HPV in the oral swab, and 49 % of these pairs were concordant for their HPV-genotype. Female partners had a higher oral HPV prevalence (16 %) than did male partners (11 %). Patients who were non-white were also found to have a higher oral prevalence of HPV (*p* = 0.032) by mouth swab.

**Conclusions:**

Partners of OPC patients may have a higher prevalence of oral HPV and should be studied prospectively to understand their OPC risk. Additional future research is needed to identify oral HPV persistence in partners to OPC patients and to determine the optimal sampling methods and technologies to screen patients at high risk for HPV-related disease.

**Electronic supplementary material:**

The online version of this article (doi:10.1186/s13027-016-0066-9) contains supplementary material, which is available to authorized users.

## Background

Human papillomavirus (HPV) is a known causative factor in most cervical premalignant and malignant conditions and in oropharyngeal squamous cell cancers [1, 2]. While genital HPV transmission between women with cervical cancer and their partners has been well studied [3], there is limited data on the rates of oral HPV transmission between patients with oropharyngeal cancer (OPC) and their partners. Even less is known about the oral transmission rate of specific HPV genotypes.

Prior cervical cancer studies have explored oral HPV transmission between female patients and their partners. A population-based Swedish study reported that husbands of wives with cervical cancer had an increased standardized incidence ratio of 2.7:1 of developing tonsillar or tongue cancer, while husbands of women with cervical intraepithelial neoplasia had a ratio of 2.4:1 [4]. Although compelling, this trial was limited by it’s retrospective nature and dependence on the Swedish Family Cancer Database, which did not account for oropharyngeal cancer risk factors such as tobacco and alcohol use. D’Souza et al. [5] recently published a prospective study analyzing oral mouth rinse samples from 98 partners of OPC patients and reported a low prevalence rate of 4.3 % oral HPV positivity.

Although the mechanism of HPV carcinogenesis is complex and involves multiple factors, it is reasonable to hypothesize that high-risk HPV genotypes are transmitted between intimate partners and that this may predispose both partners to certain HPV-related malignancies (oropharyngeal, genital, or anal). We conducted a prospective screening study of patients with OPC and their partners to evaluate the prevalence of oral HPV via a non-invasive technique, genotyped the identified oral HPV, and assessed which risk factors (i.e., alcohol and tobacco use) may be associated with increased transmission rates.

## Methods

A prospective collection of oral (oral cavity and oropharynx) epithelial cells was obtained by cytology brushing/oral mouth swab in sequentially identified newly diagnosed patients with OPC and their partners treated at MD Anderson Cancer Center (2007–2010). The eligibility criteria included informed consent from both the patient and partner, and a confirmed histologic diagnosis of oropharyngeal squamous cell cancer in the patient. Patient-partner pairs were excluded from this analysis if the patient had received any prior radiation or chemotherapy for the OPC. Also, neither the patient nor partner could have any clinical evidence of an active fungal, viral, or other infection that could compromise the oral swab results. Archived tumor tissue from prior diagnostic biopsies was collected and subjected to standard HPV testing via in situ hybridization (ISH) or p16 immunohistochemical analysis (IHC). A research assistant administered a 2-page questionnaire to the patient and partner that consisted of a detailed smoking history, prior HPV-related disease history, sexual transmitted disease infection history, and other oropharyngeal cancer risk factors, such as mouth-wash and alcohol usage. Patients and partners filled out the questionnaire at the same time but were allowed to answer the questionnaires independently and privately. The questionnaire requested information on any prior HPV-related diseases included abnormal pap smears, cervical intraepithelial neoplasia, cervical cancer, anal cancer, anogenital warts, prior head and neck cancer, OPC, or penile cancer. This study was approved by the MD Anderson institutional review board.

The primary goal of this study was to determine the prevalence of oral HPV presence, as determined by oral mouth swabs via cytology brushings, in OPC patients and their sexual partners. The secondary goals were to 1) characterize the HPV genotypes, 2) identify any risk factors that are associated with high-risk HPV oral prevalence, and 3) collect prior HPV-related disease information from partners.

For the mouth swabs, oral cytology brushes were used to gently scrape the oral mucosa at the following positions: right buccal mucosa (vertically from the high to low position), left buccal mucosa (vertically high to low position), right side of tongue, dorsal side of tongue, left side of tongue, and inside upper and lower lips. The tonsillar and base of tongue areas were then also gently scraped. If a visible tumor was present, the brush was used to gently scrape the tumor. The brush containing exfoliated cells was placed in a clearly labeled tube containing SurePath preservative fluid (<24 % denatured ethanol and 1.2 % methanol; BD Diagnostics, Tripath Imaging, Burlington, NC). The material was spun down to a cell pellet, placed in alcohol-based solution, frozen, and reserved as a back-up source of tissue specimen, if needed for analysis. Collected oral cytology specimens were frozen at –80 °C for batched HPV analysis.

DNA was extracted from SurePath Pap specimens using the DNeasy kit (catalog no. 69506, Qiagen, Valencia, CA, USA) according to the manufacturer’s instructions. Specimens with positive results on consensus primer-mediated PCR were genotyped using Easy-Chip HPV Blot (King Car Yuanshan Research Institute, I-Lan, Taiwan) as described previously [6]. First, HPV DNA was amplified by PCR assay using modified MY11/GP6t biotinylated consensus primer sets to amplify a fragment of 192 bp in the L1 open reading frame of HPV and GAPDHF/GAPDHR biotinylated primer sets were used to amplify a 136-bp segment for specimen validation as described previously [7, 8]. Briefly, PCR for HPV DNA was performed in a PCR master mixture containing 20-ng aliquot of genomic DNA, 15 mM Tris-HCl (pH 8.0), 2.0 mM MgCl_2_, 50 mM KCl, 0.25 mM each deoxynucleoside triphosphate, 0.6 mM primer, and 0.5 units of DNA polymerase (HP High Performance HotStart Taq DNA Polymerase; DNA Technologies Ltd., UK). PCR assay was performed as follows: 10 min at 95 °C, followed by 40 cycles of 30 s at 95 °C, 30 s at 45 °C, and 30 s at 72 °C and a final extension of 5 min at 72 °C. PCR for GAPDH was performed in a final reaction volume of 25 ml with a 10-ng aliquot of genomic DNA in a PCR master mixture containing 15 mM Tris-HCl (pH 8.0), 2.5 mM MgCl_2_, 50 mM KCl, 0.2 mM each deoxynucleoside triphosphate, 0.2 mM primer, and 0.5 units of HotStart Taq DNA Polymerase (DNA Technologies Ltd., UK). PCR assay was performed as follows: 10 min at 951C, followed by 40 cycles of 15 s at 95 °C, 1 min at 57 °C, and 30 s at 72 °C and a final extension of 5 min at 72 °C. A 5-ml aliquot of PCR products was analyzed by electrophoresis on a 2 % agarose gel and stained with ethidium bromide.

EasyChip HPV blot were designed to detect 39 HPV types (HPV 6, 11, 16, 18, 26, 31, 32, 33, 35, 37, 39, 42, 43, 44, 45, 51, 52, 53, 54, 55, 56, 58, 59, 61, 62, 66, 67, 68, 69, 70, 72, 74, 82, CP8061, CP8304, L1AE5, MM4, MM7 and MM8 as well as three intrinsic controls). The HPV type-specific probes were immobilized on a 14.4x9.6 mm nylon membrane, which was used for reverse-blot hybridization to detect HPV DNA in a single assay. The hybridization was performed according to the manufacturer’s instructions. Briefly, the blot membrane was equilibrated with 2x saline-sodium citrate (SCC, 1xSCC containing 0.15 M NaCl and 0.015 M sodium citrate) at room temperature for 10 min. The blot was preincubated in hybridization buffer (2xSSC, 0.5 % blocking reagent, 5 % dextran sulfate, 0.1 % sodium dodecyl sulfate (SDS), 50 mg/ml denatured salmon sperm DNA) with shaking at 35 °C for 30 min. The membrane was hybridized with 500 ml of hybridization buffer containing 20 ml of the denatured amplicons (15 ml HPV and 5 ml GAPDH PCR products) by shaking at 35 °C for at least 3 h. The blot was washed two times in washing buffer 1 (2x SSC, 0.1 % SDS) for 5 min at 251 °C and then washed two times in washing buffer 2 (0.2xSSC, 0.1 % SDS) for 5 min at 351 °C. The blot was equilibrated with buffer 1 (1x phosphate-buffered saline, pH 7.4, 0.05 % Tween 20, 0.1 % SDS) by shaking at 25 °C for 5 min and then buffer 2 (1x phosphate-buffered saline, pH 7.4, 0.05 % Tween 20, 0.1 % SDS, 0.5 % blocking reagent) at 25 °C for 1 h. The blot was incubated in 500 ml of buffer 2 containing streptavidin-AP (Calbiochem; alkaline phosphatase conjugates and biotinylated antibodies, 1:1000 dilution) at 25 °C for 40 min. The blot was then washed in buffer 1 and rinsed with buffer 3 (0.1 M Tris-HCl, pH 9.5, 0.1 M NaCl) for 5 min. Then, 80 ml NBT/BCIP (5-bromochloro- 3-indolyl-phospate and nitroblue tetrazolium) was added and incubated for 30 min at 25 °C. The reaction was terminated by adding distilled water. The HPV types were determined by visual assessment protocol provided by King Car Yuanshan Research Institute.

### Statistical methods

Chi-square or Fisher exact test was used to assess the differences in categorical variables, and the Wilcoxon rank-sum or Kruskal-Wallis test was used to detect the differences for continuous variables between the different cohorts [9]. The sensitivity and specificity of the oral swab compared to the tumor HPV status (by ISH or IHC) was calculated with exact 95 % confidence intervals (CIs). Simple kappa [9], an index of the proportion of agreement beyond that expected by chance, was used to assess the agreement of genotypes between patient-partner pairs in which both parties were HPV positive by mouth swabs. A point estimate and a 2-sided 95 % CI of the kappa were provided.

## Results

**Fig. 1:**
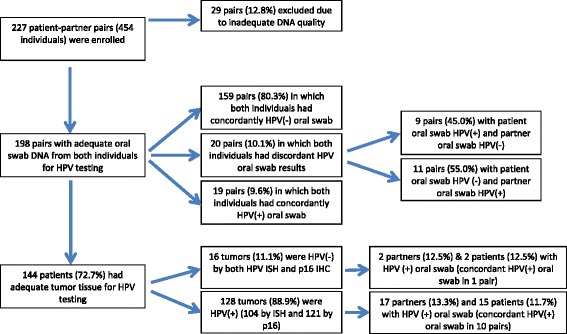
Consort diagram

This study enrolled 227 patient-partner pairs (*n* = 454 persons) from 2007 to 2010. The demographic information, exposure variables, and HPV medical history for the full cohort enrolled are presented in Additional file 1: Table S1. After DNA extraction and quality control assessment of the DNA obtained from the oral swabs, we determined that sufficient analyzable DNA was available from both parties for 198 patient-partner pairs (Fig. 1). There were no significant demographic differences between the 29 pairs (13 %) without adequate DNA and the final analyzable 198 pairs, except that the excluded pairs were older (*p* = 0.04 and *p* = 0.022, respectively; Additional file 1: Table S1). Due to lack of tumor tissue availability, only 144 OPC patients had adequate tumor tissue available for HPV testing and 128 (89 %) had HPV-positive tumors by ISH or p16 IHC (104 and 121, respectively) (Fig. 1).

In 39 of the 198 (20 %) patient-partner pairs, one or both members had an HPV-positive oral swab. In total, 28 patients (14 %) were HPV positive by oral swab, and 30 partners were HPV positive (Table 1). Partners to OPC patients had an oral HPV prevalence rate of 15 %. High-risk HPV, defined in Gillison et al. [3] was detected on oral swabs from 15 (12 %) patients with HPV-positive OPC (9 of 15 were HPV 16). Although there were only a few patients with HPV-negative tumors, a similar proportion (13 %) of patients were found to be HPV positive on oral swabs (Table 1).

**Table 1:** Prevalence of HPV in oral mouth swabs from partners and patients, stratified by tumor HPV status and sex

HPV mouth swab PCR result	All patient-partner pairs (*N* = 198)			HPV-negative tumor patient-partner pairs (*N* = 16)			HPV-positive tumor patient-partner pairs (*N* = 128)		
	Partners, *N* (%^a^)	Patients, *N* (%)	P*	Partners, *N* (%)	Patients, *N* (%)	P*	Partners, *N* (%)	Patients, *N* (%)	P*
All negative	168 (84.9)	170 (85.9)		14 (87.5)	14 (87.5)		111 (86.7)	113 (88.3)	
Oncogenic HPV positive	30 (15.2)	28 (14.1)	0.66	2 (12.5)	2 (12.5)	1.00	17 (13.3)	15 (11.7)	0.77
HPV16 positive	12 (6.1)	17 (8.6)	0.13	0 (0.0)	1 (6.3)	NA	5 (3.9)	9 (7.0)	0.13
Other HPV genotype positive	27 (13.6)	26 (13.1)	0.83	2 (12.5)	2 (12.5)	1.00	15 (11.7)	14 (10.9)	0.78
Multiple (≥2) genotypes positive	14 (7.1)	17 (8.6)	0.58	1 (6.3)	2 (12.5)	1.00	5 (3.9)	8 (6.3)	0.45
Female partners and male patients									
HPV mouth swab PCR result	All male patient-female partner pairs (*N* = 160)			HPV-negative tumor male patient-female partner pairs (*N* = 13)			HPV-positive tumor male patient-female partner pairs (*N* = 101)		
	Partners, *N* (%^a^)	Patients, *N* (%)	P*	Partners, *N* (%)	Patients, *N* (%)	P*	Partners, *N* (%)	Patients, *N* (%)	P*
All negative	134 (83.8)	137 (85.6)		11 (84.6)	11 (84.6)		85 (84.2)	88 (87.1)	
Oncogenic HPV positive	26 (16.3)	23 (14.4)	0.49	2 (15.4)	2 (15.4)	1.00	16 (15.8)	13 (12.9)	0.55
HPV16 positive	10 (6.3)	14 (8.8)	0.22	0 (0.0)	1 (7.7)	NA	5 (5.0)	8 (7.9)	0.25
Other HPV genotype positive	23 (14.4)	21 (13.1)	0.65	2 (15.4)	2 (15.4)	1.00	14 (13.9)	12 (11.9)	0.56
Multiple (≥2) genotypes positive	12 (7.5)	14 (8.8)	0.77	1 (7.7)	2 (15.4)	1.00	5 (5.0)	7 (6.9)	0.69

*McNemar test (asymptotic or exact)

^**a**^Denominators of all the percentages are the corresponding Ns in parentheses

NA = not assessed

Oral HPV16 was more commonly detected among patients than partners (9 and 6 %, respectively), and there was a strong correlation between the oral HPV status of the paired patients and partners (*p* < 0.001). However, in the partners, the oral HPV prevalence was identical between the partners of patients with HPV-positive tumors and the partners of those with HPV-negative tumors (both 13 %; Table 1 and Fig. 1). We evaluated other demographic features of the partners and discovered that female partners had a higher oral HPV prevalence (16 %) than did male partners (11 %). However, since there were only 38 male partners in this study, the small numbers did not reach statistical significance.

In the genotype analysis from the patient oral swabs, the most common genotypes were HPV 16 and HPV 56 (17 of 28 (61 %) and 14 of 28 (50 %), respectively). In the partners, HPV56 was the most common genotype detected (15 of 30 (50 %)), with HPV16 being the second most common (12 of 30 (40 %)). There were also several cases of multiple genotypes identified among both patients and partners with a positive oral swab (17 of 28 (61 %) and 14 of 30 (47 %), respectively) (Table 1).

The demographics, risk factor exposure, and HPV-related disease history of patients and partners, segregated by oral swab HPV status, are presented in Table 2. The only significant finding was that non-white patients had a higher oral swab HPV prevalence than white patients (*p* = 0.032). There were no other significant differences among patients or partners between those with and without detectable oral HPV (Table 2).

**Table 2:** Demographic data, exposure, and HPV-related disease history among participants segregated by oral swab status^f^

Characteristic	Patients (*N* = 198)			Partners (*N* = 198)		
	Swab positive (*N* = 28)	Swab negative (*N* = 170)	*p*-Value	Swab positive (*N* = 30)	Swab negative (*N* = 168)	*p*-value
Age, years						
< 55	12 (42.9 %)	76 (44.7 %)	.86	15 (50 %)	93 (56 %)	.54
≥ 55	16 (57.1 %)	94 (55.3 %)		15 (50 %)	73 (44 %)	
Sex						
Female	5 (17.9 %)	32 (18.8 %)	1.0	26 (86.7 %)	135 (80.4 %)	.61
Male	23 (82.1 %)	138 (81.2 %)		4 (13.3 %)	33 (19.6 %)	
Race						
White	22 (78.6 %)	156 (91.8 %)	.032	23 (76.7 %)	134 (79.8 %)	.70
Other	6 (21.4 %)	14 (8.2 %)		7 (23.3 %)	34 (20.2 %)	
Smoking status^a^						
Former/never	14 (70.0 %)	80 (71.4 %)	.90	13 (61.9 %)	61 (67.8 %)	.61
Current	6 (30 %)	32 (28.6 %)		8 (38.1 %)	29 (32.2 %)	
Prior alcohol use^b^						
None	3 (11.1 %)	15 (8.9 %)	.72	6 (20 %)	34 (21.1 %)	.89
Yes	24 (88.9 %)	154 (91.1 %)		24 (80 %)	127 (78.9 %)	
Prior HPV-related disease						
None	28 (100.0 %)	157 (93.5 %)	.37	23 (76.7 %)	133 (80.1 %)	.67
Yes	0 (0.0 %)	11 (6.5 %)		7 (23.3 %)	33 (19.9 %)	
Prior benign HPV-related disease^c^						
None	28 (100.0 %)	165 (97.1 %)	1.0	30 (100 %)	161 (95.8 %)	.60
Yes	0 (0.0 %)	5 (2.9 %)		0 (0 %)	7 (4.2 %)	
Prior HPV-related malignancy^d^						
None	27 (96.4 %)	165 (97.1 %)	1.0	29 (96.7 %)	163 (97 %)	1.0
Yes	1 (3.6 %)	5 (2.9 %)		1 (3.3 %)	5 (3 %)	
HPV status of oropharyngeal tumor^e^						
Negative	2 (11.8 %)	14 (11 %)	1.0	2 (10.5 %)	14 (11.2 %)	1.0
Positive	15 (88.2 %)	113 (89 %)		17 (89.5 %)	111 (88.8 %)	
Oral mouth swab status of matched pair						
HPV Negative	9 (32.1 %)	159 (93.5 %)	<.001	11 (36.7 %)	159 (94.6 %)	<.001
HPV Positive	19 (67.9 %)	11 (6.5 %)		19 (63.3 %)	9 (5.4 %)	

^a^8 swab positive and 58 swab negative patients had missing data on smoking; 1 swab positive and 1 swab negative patient had missing data on prior alcohol use

^b^9 swab positive and 78 swab negative partners had missing data on smoking; 7 swab negative partners had missing data on prior alcohol use

^c^benign HPV-related disease defined as CIN I-III, dysplasia

^d^Cervical cancer, anal cancer, prior HNSCC cancer

^e^By p16 IHC (*N* = 121) or HPV ISH (*N* = 104); data were unavailable for 54 patients

^f^Percentages are calculated as a column percentage

Table 3 presents the concordance rates of HPV status between patient and partner pairs (i.e., both positive, both negative, or discordant) and is also segregated by HPV genotype. Nineteen couples had both members demonstrate an HPV-positive oral swab (Table 2). Within these 19 couples, genotype analysis showed that 11 (58 %) were both positive for HPV16, eight (42.1 %) were both positive for HPV 56 (four in conjunction with HPV16), and nine were positive for multiple HPV genotypes (Table 3). The genotype results did not significantly change when evaluating couples where the patient had a documented HPV-positive tumor or when assesing those couples with a female partner (Table 3). However, in couples who both had HPV-positive oral swabs, partners were more likely to be older age ≥ 55 years than partners with concordant negative results (68 % vs 42 %; *p* = 0.03; Table 4). In addition, couples who were both HPV positive by oral swabs were more likely to consist of a non-white patient than couples with concordant negative results (32 % vs 8 %, *p* = 0.002; Table 4). There were no other significant differences in the distribution of demographics, exposure, or HPV-related disease history between couples with concordant positive and concordant negative oral HPV prevalence.

**Table 3:** Concordant prevalence in patient-partner pairs for oncogenic HPV and genotypes with segregation by tumor HPV status and sex*

HPV swab PCR result	All patient-partner pairs (*N* = 198)			HPV-negative tumor patient-partner pairs (*N* = 16)			HPV-positive tumor patient-partner pairs (*N* = 128)		
	Concordant +, *N* (%)	Concordant -, *N* (%)	Discordant, *N* (%)	Concordant +, *N* (%)	Concordant -, *N* (%)	Discordant, *N* (%)	Concordant +, *N* (%)	Concordant -, *N* (%)	Discordant, *N* (%)
High-risk HPV	19 (9.6)	159 (80.3)	20 (10.1)	1 (6.3)	13 (81.3)	2 (12.5)	10 (7.8)	106 (82.8)	12 (9.4)
HPV16	11 (5.6)	180 (90.9)	7 (3.5)	0 (0.0)	15 (93.8)	1 P (6.3)	5 (3.9)	119 (93.0)	4 (3.1)
HPV18	2 (1.0)	195 (98.5)	1 (0.5)	1 (6.3)	15 (93.8)	0 (0.0)	1 (0.8)	127 (99.2)	0 (0.0)
HPV31	0 (0.0)	196 (99.0)	2 (1.0)	0 (0.0)	15 (93.8)	1 P (6.3)	0 (0.0)	127 (99.2)	1 (0.8)
HPV39	0 (0.0)	197 (99.5)	0 (0.0)	1 (6.3)	15 (93.8)	0 (0.0)	0 (0.0)	128 (100.0)	0 (0.0)
HPV43	0 (0.0)	197 (99.5)	1S (0.5)	0 (0.0)	16 (100)	0 (0.0)	0 (0.0)	127 (99.2)	1 (0.8)
HPV45	0 (0.0)	193 (97.5)	5 (2.5)	0 (0.0)	16 (100)	0 (0.0)	0 (0.0)	126 (98.4)	2 (1.6)
HPV51	0 (0.0)	197 (99.5)	1 P (0.5)	0 (0.0)	16 (100)	0 (0.0)	0 (0.0)	128 (100.0)	0 (0.0)
HPV52	0 (0.0)	195 (98.5)	3 S (1.5)	0 (0.0)	16 (100)	0 (0.0)	0 (0.0)	126 (98.4)	2 (1.6)
HPV54	0 (0.0)	195 (98.5)	3 (1.5)	0 (0.0)	16 (100)	0 (0.0)	0 (0.0)	126 (98.4)	2 (1.6)
HPV56	8 (4.0)	177 (89.4)	13 (6.6)	0 (0.0)	14 (87.5)	2 (12.5)	5 (3.9)	115 (89.8)	8 (6.2)
HPV59	2 (1.0)	194 (98.0)	2 (1.0)	0 (0.0)	16 (100)	0 (0.0)	1 (0.8)	126 (98.4)	1 (0.8)
HPV61	0 (0.0)	195 (98.5)	3 (1.5)	0 (0.0)	16 (100)	0 (0.0)	0 (0.0)	126 (98.4)	2 (1.6)
HPV67	1 (0.5)	194 (98.0)	3 (1.5)	0 (0.0)	16 (100)	0 (0.0)	0 (0.0)	126 (98.4)	0 (0.0)
HPV70	0 (0.0)	197 (99.5)	1 S (0.5)	0 (0.0)	16 (100)	0 (0.0)	0 (0.0)	128 (100.0)	0 (0.0)
HPV84	0 (0.0)	195 (98.5)	3 S (1.5)	0 (0.0)	0 (0.0)	0 (0.0)	0 (0.0)	128 (100.0)	0 (0.0)
Multiple types (≥2)	9 (4.6)	176 (88.9)	13 (6.6)	1 (6.3)	14 (87.5)	1 (6.3)	3 (2.3)	118 (92.2)	7 (5.5)
Female partners of male patients									
	*N* = 160			*N* = 13			*N* = 101		
	Concordant +, *N* (%)	Concordant -, *N* (%)	Discordant, *N* (%)	Concordant +, *N* (%)	Concordant -, *N* (%)	Discordant, *N* (%)	Concordant +, *N* (%)	Concordant -, *N* (%)	Discordant, *N* (%)
High-risk HPV	15 (9.4)	126 (78.8)	19 (11.9)	1 (7.7)	10 (76.9)	2 (15.3)	9 (8.9)	81 (80.2)	11 (10.9)
HPV16	9 (5.6)	145 (90.6)	6 (3.8)	0 (0.0)	12 (92.3)	1 P (7.7)	5 (5.0)	93 (92.1)	3 (3.0)
Other HPV types	12 (7.5)	128 (80.0)	20 (12.58)	1 (7.7)	10 (76.9)	2 (15.3)	7 (6.9)	82 (81.2)	12 (11.9)
Multiple types (≥2)	7 (4.4)	141 (88.1)	12 (7.5)	1 (7.7)	11 (84.6)	1 (7.7)	3 (3.0)	92 (91.1)	6 (5.9)

*Denominators of the percentages are the corresponding Ns in the parentheses

S indicates partner/spouse; P indicates patient

**Table 4:** Demographics, exposure, and HPV-related disease history of HPV concordant positive and negative pairs

Characteristic	All patient-partner pairs+ (*N* = 198)		
	Concordant positive (*N* = 19)	Concordant negative (*N* = 159)	*p*-value*
Patient age, years			
< 55	7 (36.8 %)	69 (43.4 %)	.59
≥ 55	12 (63.2 %)	90 (56.6 %)	
Partner age, years			
< 55	6 (31.6 %)	91 (57.6 %)	.03
≥ 55	13 (68.4 %)	67 (42.4 %)	
Patient sex			
F	4 (21.1 %)	32 (20.1 %)	1.0
M	15 (78.9 %)	127 (79.9 %)	
Partner sex			
F	15 (78.9 %)	127 (79.9 %)	1.0
M	4 (21.1 %)	32 (20.1 %)	
Patient race			
White	13 (68.4 %)	146 (91.8 %)	.002
Other	6 (31.6 %)	13 (8.2 %)	
Partner race			
White	13 (68.4 %)	125 (78.6 %)	.31
Other	6 (31.6 %)	34 (21.4 %)	
Patient smoking status			
Former/never	8 (61.5 %)	76 (71.7 %)	.52
Current	5 (38.5 %)	30 (28.3 %)	
Partner smoking status			
Former/never	7 (53.8 %)	58 (66.7 %)	.37
Current	6 (46.2 %)	29 (33.3 %)	
Patient history of benign HPV disease			
None	19 (100 %)	154 (96.9 %)	1.0
Yes	0 (0 %)	5 (3.1 %)	
Partner history of benign HPV disease			
None	19(100 %)	153 (96.2 %)	1.0
Yes	0 (0 %)	6 (3.8 %)	
Patient history of HPV cancer			
None	18 (94.7 %)	155 (97.5 %)	.44
Yes	1 (5.3 %)	4 (2.5 %)	
Partner history of HPV cancer			
None	19 (100 %)	155 (97.5 %)	1.0
Yes	0 (0 %)	4 (2.5 %)	

*Fisher’s exact test

+Percentages are all column percentages

## Discussion

In this study of almost 200 OPC patients and their partners, we identified an oral HPV prevalence rate of 15 % among partners by oral swab sampling and a14 % rate for patients. A high proportion (89 %) of OPC tumors were positive for HPV when tested by HPV-ISH or p16 IHC, consistent with the results of a prior large contemporary series from our institution [10]. In our patient population, oral swab HPV testing had a low sensitivity for determining HPV tumor status. We suspect that rather than detecting tumor HPV, we are detecting prevalent HPV infections being shared between patients and partners. This is supported by the fact that we detected oral HPV in approximately two-thirds of the partners of HPV-positive oral mouth swab patients.

The oral HPV prevalence in partners (15 % overall and 16 % for female partners) was higher than that previously reported for the general population (7 and 4 % for women) using National Health and Nutrition Examination Survey (NHANES) 2009–2010 [3] and in a recently reported study of partners of OPC patients (4 and 2 % for female partners) [5]. This discrepancy could potentially be explained by several reasons. First, our sample size of partners was larger (*n* = 198) than that (*n* = 93) recently reported by D’Souza et al [5]. Thus, we may have a more stable estimate of oral HPV prevalence. Second, our patient population may have had demographic differences that are linked to a higher oral HPV prevalence. We did exclude 29 couples due to inadequate DNA quality by oral mouth swab, and these individuals were slightly older. However, of note, the median age of our partners (age 53) was identical to that in the D’Souza study. Our study did include a higher number of male partners (19 %) and non-white partners (21 %) than the D’Souza study (7 and 6 %, respectively). We also had a higher number of partners who were current smokers and drinkers (33 % and 79 %, respectively) compared to the D’Souza et al. study, 12 % current smokers and 65 % alcohol drinkers. Both of these behaviors may be associated with more high-risk sexual practices. In addition, we reported a higher rate of prior HPV-related disease among female partners (25 %) than that identified in the D’Souza study (10 %) [5]. Unfortunately, a significant limitation of our study was that we lacked clinical data on sexual practices and lifetime oral and genital sex partners, which could have also contributed to the higher prevalence of oral HPV. Third, our detection method, using an oral mouth swab with a cytology brush and swabbing eight or nine areas within the oral cavity and oropharynx, may have been more aggressive than those used in prior reports [3, 5] where oral rinses were typically used.

Our results suggest that partners of OPC patients have a higher oral HPV prevalence than that found in the general population [3]. However, whether this higher oral HPV prevalence will ultimately translate into a significantly higher risk of HPV-associated OPC is unknown and requires additional study. Dalla Torre et al. [11] has previously reported that high-risk oral HPV infections are associated with the presence of oral premalignant lesions. It can be extrapolated that since persistent cervical HPV status is directly linked to a higher risk of progression to high-grade cervical intraepithelial neoplasia and cervical cancer, it is critical that we understand the natural history of oral HPV infections and investigate the significance of persistent oral infections.

High oral HPV16 load has been reported to be associated with a longer time to clearance of infection [12]. In the HPV Infection in Men (HIM) cohort study [13], which evaluated 1626 men over 1 year, newly acquired oral HPV16 infections were identified in 0.6 % (*n* = 18), and most of the infections had cleared after 1 year. However, eight of these men had persistent infections for the entire study duration. A population with persistently detected oral HPV would be an ideal group in which to study novel screening technologies.

One of the issues that requires more investigation is identifying the optimal screening method. Kreimer et al. [14] reported that blood HPV16 E6 seropositivity was identified in 35 % of OPC patients and was found more than 10 years prior to the cancer diagnosis. Rettig et al. [15] evaluated 124 definitively treated HPV-positive OPC patients and obtained oral rinse and gargle samples at 9, 12, 18, and 24 months after therapy. They reported that five patients with persistent oral HPV16 status had poorer disease-free survival (more local recurrence) and overall survival. However, while the specificity of the oral rinse and gargle was 100 %, the sensitivity was only 43 %; suggesting that the optimal screening method remains unknown. Taken together with this growing body of literature [3, 16, 17], the results of our study suggest that the method of sampling is important and that oral mouth swabs with aggressive sampling are a better methodology to be explored with other testing strategies, such as serology [14, 18] or potentially ultrasound [19] evaluation.

## Conclusion

Partners of OPC patients may have a higher prevalence of oral HPV and should be studied prospectively to refine our understanding of OPC risk among these individuals. Future research is needed to determine oral HPV persistence in partners, optimal sampling methods for oral HPV, and possible technologies for screening these patients who are at higher risk for HPV-related disease.

## Additional file

Additional file 1: Table S1. Demographics, exposure, and HPV-related disease history among all evaluable participants enrolled versus those who were excluded from the final analysis. (DOCX 16 kb)
