# Supplementary material for: Concordance of oral HPV prevalence between patients with oropharyngeal cancer and their partners
Source: Infect Agent Cancer. 2016 Apr 27;11:21. doi: 10.1186/s13027-016-0066-9 (PMC4847345; doi:10.1186/s13027-016-0066-9)
Supplement: Additional file 1: Table S1. — Demographics, exposure, and HPV-related disease history among all evaluable participants enrolled versus those who were excluded from the final analysis. (DOCX 16 kb) [file 13027_2016_66_MOESM1_ESM.docx]

**Additional file 1: Table S1. Demographics, exposure, and HPV-related disease history among all evaluable participants enrolled versus those who were excluded from the final analysis.**

| **Characteristics** | **Patients (n=227)** | | | **Partners (N=227)** | | |
| --- | --- | --- | --- | --- | --- | --- |
|  | **Evaluable (n=198)** | **Excluded (n=29)** | **p-value^*^** | **Evaluable (n=198)** | **Excluded (n=29)** | **p-value^*^** |
| Age, years | | | | | | |
| <55 | 88 (44.4%)^+^ | 7 (24.1%) | .038 | 109 (55.3%) | 9 (32.1%) | .022 |
| ≥55 | 110 (55.6%) | 22 (75.9%) |  | 88 (44.7%) | 19 (67.9%) |  |
| Sex | | | | | | |
| Female | 37 (18.7%) | 5 (17.2%) | 1.0 | 161 (81.3%) | 24 (82.8%) | 1.00 |
| Male | 161 (81.3%) | 24 (82.8%) |  | 37 (18.7%) | 5 (17.2%) |  |
| Race | | | | | | |
| White | 178 (89.9%) | 25 (86.2%) | .52 | 157 (79.3%) | 26 (89.7%) | .22 |
| Other | 20 (10.1%) | 4 (13.8%) |  | 41 (20.7%) | 3 (10.3%) |  |
| Smoking status | | | | | | |
| Former/never | 94 (71.2%) | 15 (71.4%) | 0.98 | 74 (66.7%) | 15 (75%) | 0.61 |
| Current | 38 (28.8%) | 6 (28.6%) |  | 37 (33.3%) | 5 (25%) |  |
| Alcohol usage | | | | | | |
| None | 18 (9.2%) | 1 (3.4%) | .48 | 41 (21.4%) | 6 (20.7%) | .94 |
| Yes | 178 (90.8%) | 28 (96.6%) |  | 151 (78.6%) | 23 (79.3%) |  |
| Prior HPV disease | | | | | | |
| None | 185 (94.4%) | 27 (93.1%) | .68 | 156 (79.6%) | 24 (82.8%) | .81 |
| Yes | 11 (5.6%) | 2 (6.9%) |  | 40 (20.4%) | 5 (17.2%) |  |
| Prior benign HPV disease | | | | | | |
| None | 193 (97.5%) | 29 (100%) | 1.0 | 191 (96.5%) | 28 (96.6%) | 1.0 |
| Yes | 5 (2.5%) | 0 (0%) |  | 7 (3.5%) | 1 (3.4%) |  |
| Prior HPV-related cancer | | | | | | |
| None | 192 (97%) | 26 (89.7%) | .093 | 192 (97%) | 29 (100%) | 1.0 |
| Yes | 6 (3%) | 3 (10.3%) |  | 6 (3%) | 0 (0%) |  |
| Prior tonsillectomy | | | | | | |
| Never | 112 (57.7%) | 14 (50%) | .44 | 130 (68.4%) | 20 (69%) | .95 |
| Yes | 82 (42.3%) | 14 (50%) |  | 60 (31.6%) | 9 (31%) |  |
| HPV status of oropharyngeal tumor | | | | | | |
| Negative | 16 (11.1%) | NA | NA | 16 (11.1%) | NA | NA |
| Positive | 128 (88.9%) | NA |  | 128 (88.9%) | NA |  |

*Fisher’s exact test.

^+^Column percentage.

NA=not assessed.
